# Setting up and functioning of a preanaesthetic clinic

**DOI:** 10.4103/0019-5049.72638

**Published:** 2010

**Authors:** Anju Gupta, Nishkarsh Gupta

**Affiliations:** Department of Anaesthesiology and Intensive Care, Lady Hardinge Medical College, New Delhi - 110 001, India; 1 Department of Anesthesia and Pain Management, PGIMER and Associated Dr. RML Hospital, Chandigarh, India

**Keywords:** Benefits, preanaesthesia clinics, setup

## Abstract

The goal of preoperative risk assessment is to identify and modify the procedure and patient factors that significantly increase the risk for complications. Preanaesthesia clinics (PACs) have been developed to improve the preoperative experience of the patients by coordinating surgical, anaesthesia, nursing and laboratory care. These clinics can also help in developing practice guidelines, and decreasing the number of consultations, laboratory tests and surgical cancellations. Though these clinics are present in most of our hospitals, a major effort is needed to upgrade these setups so as to maximise the benefits. This review gives a brief account of organisation and functioning of PACs.

## INTRODUCTION

Preanaesthesia evaluation is the process of clinical assessment by an anaesthetist, which precedes the delivery of anaesthesia care for surgery and non-surgical procedures.[1] Preanaesthetic clinic (PAC) is a specialty clinic where patients are evaluated before surgery to establish a database upon which risk assessment and perioperative management decisions can be made. It includes an interview and examination of the patient, a review of previous medical, surgical and anaesthesia problems, a detailed account of current medication use, and provisions for obtaining and reviewing preoperative tests.

The goals are to:[1,2]

1. create a rapport with the patients and their families and allay their anxiety;
2. reduce the morbidity of surgery;
3. increase the quality;
4. reduce surgical delays and case cancellations and
5. decrease the cost of preoperative care.

Traditionally, elective surgical patients were admitted to hospital the day before surgery to undergo preanaesthetic assessment, risk optimisation and preoperative preparation. This practice is no longer a routine in many parts of the world because of its lack of cost-effectiveness. In addition, in-patient evaluation did not effectively eliminate day-of-surgery cancellations due to inadequate optimisation of co-morbidities[3] and administrative factors.

Moreover, as the focus of health care delivery has been recently towards ambulatory care, an efficient working PAC is required.

## LOCATION OF PREANAESTHESIA CLINICS

It can be situated in the same hospital complex as other surgical specialty clinics to promote easy accessibility and convenience. Moreover, this may allow patients to be assessed at the PAC on the same day as their surgical appointments.

The main parameters for an ideal PAC location are the following.

1. It should be easily accessible from the main entrance of the hospital.
2. Preferably, it should be in the main out patient department (OPD) block near surgical specialties.
3. It should be separate from the in-patient facility.
4. It should be preferably on the ground floor.
5. There should be an easy access to the hospital diagnostic and other support facilities (waiting area, safe drinking water and toilets for patients and their relatives).

The physical design of the clinic should provide adequate space demarcated into areas for

- registration and reception,
- patient interview and examination,
- patient and family preoperative education and
- staff rest.

The PAC should provide a relaxed and private atmosphere for the following activities.[4]

1. Preanaesthesia evaluation through review of the medical records, history, examination and relevant ancillary testing, followed by risk optimisation through appropriate interventions and consultations.
2. Discussion of the risks and benefits of anaesthetic options and pain management strategies.
3. Alleviation of anxiety through counselling.
4. Patient and family education on topics such as fasting, medications to continue on the day of surgery, special nursing care requirements, anticipated duration of hospital stay, transportation issues and contingency for undercurrent illness.
5. Validation of consent and documentation of advanced medical directives (if any).
6. Reduction of day-of-surgery delay or no-show via telephone calls made on the day before surgery.

## STAFFING REQUIREMENTS

A senior specialist/consultant should be responsible for policy administration and quality assurance.

Medical officers as well as trainees’ in anaesthesia should be posted to the clinic as part of their training to ensure training in preanaesthesia evaluation and optimisation. Any queries regarding the optimisation and fitness of the patient should be discussed with the consultant posted. Any case found to have a American Society of Anesthesiologist (ASA) status score of >II should also be discussed with the senior anaesthesiologist involved in giving anaesthesia, so as to avoid last minute cancellations.

## APPOINTMENTS

In general, patients are screened at the PAC from 2 to 30 days or more before their scheduled date of surgery.[1] There are not enough data in the literature on the optimal timing for preanaesthesia evaluation. Factors that should be used to guide the timing of preanaesthesia evaluation are patient demographics and clinical conditions, the type and invasiveness of the procedure, and availability of resources provided by the specific practice environment. ASA Task Force has recommended that preanaesthesia evaluations should be performed prior to the day of surgery for patients with high severity of disease and/or undergoing procedures of high surgical invasiveness.[1]

Scheduling should also ensure an even patient flow, the timely reporting of results by laboratory and diagnostic imaging services, a system of medical referral for timely optimisation and an efficient appointment system.

## DATA COLLECTION AND RECORDING

The basic information about the patients, the surgery planned, past medical and surgical history can be obtained with the help of a specially designed questionnaire. This can be distributed to the patients in the waiting area and filled by them with the help of relatives/staff nurses. Such practices are widely prevalent in other countries and should be enforced in our country. This will reduce the load on the anaesthetists and will go a long way in improving the overall functioning of the PAC clinic.

A computer database of the details of the preanaesthtic check-up of the patients can be made so that it can be reviewed by anybody connected to the network. Such electronic medical records allow standardisation of patient information, avoid redundancy, and provide a database for research.

## BENEFITS OF AN EFFECTIVE FUNCTIONING PREANAESTHETIC CLINIC

### Reduction in excessive preoperative testing

The ASA Task Force recommends that preoperative tests may be ordered, required or performed *on a selective basis* for purposes of guiding or optimising perioperative management. The indications for such testing should be documented and based on information obtained from medical records, patient interview, physical examination, and type and invasiveness of the planned procedure. A test should be ordered only if it can correctly identify abnormalities and will change the diagnosis, the management plan or the patient’s outcome.

It has been estimated that 60–75% of preoperative tests ordered are medically unnecessary.[5–7] Indiscriminate testing can increase the risk of iatrogenic injury arising from unnecessary testing or treatment when a borderline or false-positive result is obtained.

A false-positive result distracts the physician from detecting or pursuing a clinically more significant problem and may eventually harm the patient. Furthermore, unnecessary testing may cause a delay or cancellation of the planned surgery. It is better not to order an unnecessary test because the medico-legal risk is greater for not following an abnormal test result than for not ordering a test that was not indicated. Hiding clinically insignificant abnormal laboratory test finding can result in legal action if it is not evaluated further.

PACs should help hospitals to standardise and optimise preoperative laboratory testing. A protocol based on scientific evidence and local practices should be formulated and circulated among the operating surgeons and the anaesthetists. The surgeons should refer the patients to the PAC with the preoperative testing based on the protocol so as to minimise the delay in their surgery.

## REDUCTION IN SUBSPECIALTY CONSULTS

Preanaesthesia evaluation consists of the consideration of information from multiple sources which may include the patient’s medical records, interview, physical examination, and findings from medical tests and evaluations. A thoroughly conducted PAC can improve the safety and effectiveness of anaesthetic processes involved with perioperative care by optimising the preoperative co-morbid conditions. Every effort should be done to minimise the unnecessary subspecialty consults which may include interventions that result in injury, discomfort, inconvenience, delays or costs that are not commensurate with the anticipated benefits.

A PAC can reduce the use of costly subspecialty consults without affecting patient outcome. The implementation of more stringent consultation algorithms through a high volume, tertiary care PAC led to a significantly reduced rate of preoperative cardiology consultations.[8] Alternatively, having a PAC staffed by physicians who are trained in both internal medicine and anaesthesia can further enhance quality patient care with hospital cost savings.

### Enhanced operative room functioning

PACs have a positive impact on effective functioning of the operating room. Preoperative risk assessment can only be accomplished if adequate knowledge of co-morbid conditions is obtained with the help of old medical records, test results and notes from other hospitals at the time of the preoperative evaluation. Optimisation of preexisting/recently diagnosed medical conditions plays a major role in reducing the cancellations and delays on the day of surgery.[9]

Delay or cancellation within 24 hours of planned surgery is highly undesirable as it causes distress to the patient, disrupts bed management, reduces operating room efficiency and increases costs incurred by having to maintain a facility that is essentially not generating productivity. Several studies have found significant reduction in the cancellation rates after implementation of outpatient preanaesthesia evaluation services.[10–12]

Fischer reported a decrease in the rate of day-of-surgery cancellation from 1.96% in the year before implementation of the anaesthesia preoperative evaluation clinic to 0.21% in the year following its implementation at the Stanford University Hospital.

Cancelled cases may delay subsequent cases and waste expensive case setups. By reducing case cancellations, a PAC can improve operating room efficiency on the day of surgery and have a significant financial benefit to a hospital with a busy operating room schedule. This becomes more relevant in our country because of limited resources available.

### Preanaesthesia evaluation and day care surgery

Ambulatory surgery is being practiced widely throughout the world. Out-patient preanaesthesia assessment needs to keep pace with the increasing number and complexity of the ambulatory surgery population. Controversies exist with regard to the timing of preoperative evaluation and the need for out-patient PAC assessment.[13] There is no strong evidence in the literature on the optimal timing for PAC. Traditionally, patients undergo PAC screening 1–30 days before and are admitted a day prior to the scheduled day of surgery. In order to maintain efficiency and patient safety while minimising delays and cancellations, out-patients posted for day care surgeries are now routinely screened preoperatively using several methods. These include questionnaires, telephone interviews,[14] automated interviews, and evaluation at a preanaesthesia assessment clinic. This information identifies potential problems (medical, anaesthetic or social), helps to triage patients according to the risk involved, and order relevant laboratory tests and consultations.[15,16] This also decreases the workload of the PAC and reduces the number of hospital visits. So, there is a reduction in the day-of-surgery delays/cancellations and the overall cost, which is of utmost importance in the day care surgery.

### Preanaesthesia clinics and telemedicine technology

The internet-based health solutions (telemedicine) have a substantial impact on health care and are used in medical and surgical specialties since many years.[17] There is recently an increased interest in utilising telemedicine technology for preadmission anaesthesia consultations. The patient does not have to travel for routine consultations, and moreover, if an expert consultation is required in case of a comorbid disease, it can be achieved immediately via teleconferencing in the presence of an anaesthetist.[18] This reduces the time spent for super specialist consultations and the extra investigation which would be ordered otherwise. The patients’ estimated cost and time involved in telemedicine consultation is lesser as compared to a conventional preoperative anaesthesia consultation. The format of the telemedicine anaesthesia consultation has been found to be satisfactory for the patient, the consulting and attending anaesthetists. So, telemedicine is a boon to health care system and providers and helps them provide a better coordinated and quality care to the patients.

## LIMITING FACTORS

The most limiting factors for implementation of a functioning PAC are lack of finance and shortage of anaesthetists to run the clinic. Lack of finance is a frequently reported problem, especially in a private setup where the anaesthetists often work on a fee per case basis. The overall benefits are of a greater magnitude than the limitations perceived by us. With the motivation and cooperation of the anaesthetists, a PAC can be established even with limited resources.

## CONCLUSION

A thorough preoperative evaluation can be as effective as an anxiolytic premedication. All the departments of anaesthesia should have guidelines regarding timing, preoperative testing and super speciality consultations for effective functioning of a PAC to reduce the number of visits. These guidelines should be continuously updated and made available online to all providers within the institution. Efforts should be made to utilise the upcoming telemedicine technology in our preanaesthesia consultations also.
